# Pediatric Awake Craniotomy Within a Structured Perioperative Pathway: A Case Report of Anesthesiologic Management for Recurrent Astrocytoma in a 10-Year-Old Child

**DOI:** 10.3390/children13070916

**Published:** 2026-07-10

**Authors:** Francesco Smedile, Dario Cirillo, Alessandro Vittori, Mariangela Padua, Andrea Carai, Alessandra Savioli, Antonella Cacchione, Alessandro De Benedictis, Rosanna Pariante, Corrado Cecchetti

**Affiliations:** 1 Department of Anesthesia, Critical Care and Pain Medicine, ARCO, Bambino Gesù Children’s Hospital (IRCCS), Piazza S. Onofrio 4, 00165 Rome, Italy; francesco.smedile@opbg.net (F.S.); alexvittori@libero.it (A.V.); mariangela.padua@opbg.net (M.P.); alessandra.savioli@opbg.net (A.S.); rosanna.pariante@opbg.net (R.P.); 2 Department of Neurosciences, Reproductive and Odontostomatological Sciences, University of Naples “Federico II”, Via Pansini, 5, 80131 Naples, Italy; 3 Neurosurgery Unit, Department of Neurosciences, Bambino Gesù Children’s Hospital (IRCCS), Piazza S. Onofrio 4, 00165 Rome, Italy; andrea.carai@opbg.net (A.C.); alessandro.debenedictis@opbg.net (A.D.B.); 4 Department of Onco-Hematology, Cell Therapy, Gene Therapy, and Hemopoietic Transplant, Bambino Gesù Children’s Hospital (IRCCS), Piazza S. Onofrio 4, 00165 Rome, Italy; antonella.cacchione@opbg.net; 5 Pediatric Intensive Care Unit, Department of Emergency, Acceptance and General Pediatrics, Bambino Gesù Children’s Hospital (IRCCS), Piazza S. Onofrio 4, 00165 Rome, Italy; corrado.cecchetti@opbg.net

**Keywords:** awake craniotomy, pediatric neuroanesthesia, language mapping, dexmedetomidine, asleep–awake–asleep, constitutional mismatch repair deficiency, eloquent cortex, case report

## Abstract

**Highlights:**

**What are the main findings?**

- Pediatric awake craniotomy with intraoperative language mapping was safely completed in a 10-year-old with constitutional mismatch repair deficiency (CMMRD) and a recurrent eloquent-area astrocytoma, using an asleep–awake–asleep strategy.
- A controlled airway approach (supraglottic device plus fiberoptic-guided intubation) combined with low-dose dexmedetomidine allowed cooperation during mapping without major adverse events.

**What are the implications of the main findings?**

- Age alone should not exclude children from awake craniotomy; suitability depends on developmental maturity, communication skills, and family support.
- Success depends on a structured multidisciplinary perioperative pathway rather than on any single intraoperative technique.

**Abstract:**

**Background:** Awake craniotomy is well established in adult neurosurgery for lesions near eloquent cortical areas, but its use in children remains uncommon and presents substantial anesthetic, psychological, and organizational challenges. We report the anesthesiologic management of awake craniotomy in a child with constitutional mismatch repair deficiency (CMMRD). **Case Presentation:** A 10-year-old boy with CMMRD underwent resection of a recurrent left frontal IDH-mutant astrocytoma, WHO grade 3, located adjacent to eloquent language cortex. Because of the high risk of postoperative language impairment, a structured multidisciplinary pathway was implemented, including neurosurgical, neuropsychological, and anesthesiologic evaluation, preoperative familiarization, and intraoperative language testing. An asleep–awake–asleep strategy was used. After induction with propofol, fentanyl, and rocuronium, the airway was secured with a supraglottic airway device (air-Q) followed by fiberoptic-guided tracheal intubation. During the awake phase, propofol was discontinued, remifentanil reduced, and low-dose dexmedetomidine introduced to preserve cooperation. The patient completed intraoperative language mapping, enabling identification of functional language boundaries and safe resection. No major intraoperative adverse events, airway complications, or seizures occurred. Postoperative pain scores remained below 4 on an age-appropriate Numeric Rating Scale, and the patient showed no new neurological deficits. At 1-month follow-up he remained symptom-free. **Conclusions:** This case adds to the still limited pediatric experience with awake craniotomy and suggests that, in carefully selected children managed within a structured multidisciplinary perioperative pathway and with a tailored anesthetic strategy, the procedure can be performed safely; confirmation in larger series is nonetheless required.

## 1. Introduction

Awake craniotomy is a well-established technique in adult neurosurgery for lesions located near eloquent cortical areas, allowing intraoperative functional mapping to maximize tumor resection while minimizing neurological deficits [1]. Although still relatively uncommon in the pediatric population, growing evidence suggests that this approach can be feasible and safe in carefully selected patients; in this setting, the main challenge extends beyond the surgical indication itself and includes perioperative anesthetic management [2,3]. Most reported cases involve children between 7 and 17 years of age, who generally have sufficient cognitive and emotional maturity to cooperate during intraoperative testing, with supratentorial tumors—particularly gliomas—representing the most frequent indication, followed by epilepsy surgery and, less commonly, vascular lesions [2,3,4].

Achieving an appropriate balance among sedation, analgesia, airway safety, emotional comfort, and active cooperation during cortical mapping is particularly demanding in children [3,4,5]. Several anesthetic strategies have been described, most commonly based on an asleep–awake–asleep approach combined with scalp nerve blocks, local anesthetic infiltration, and titratable sedative agents such as dexmedetomidine [3,6]. In addition to intraoperative management, successful implementation requires careful multidisciplinary planning, including patient selection, preoperative psychological preparation, and familiarization with the operating room environment [2,3,4,5,7].

Despite increasing interest in pediatric awake craniotomy, the available literature remains limited and is largely based on case series and single-case reports [2,3,4]. Detailed reports focusing specifically on perioperative anesthetic management are still scarce, particularly in complex clinical scenarios. Here we report the anesthesiologic management of awake craniotomy in a 10-year-old child with constitutional mismatch repair deficiency (CMMRD), a rare genetic syndrome associated with a high predisposition to multiple malignancies, who underwent resection of a recurrent isocitrate dehydrogenase (IDH)-mutant astrocytoma located adjacent to eloquent language cortex.

## 2. Case Presentation

Written informed consent for publication of this case and accompanying images was obtained from the patient’s parents. Publication of this case report was approved by the Ethics Committee of Bambino Gesù Children’s Hospital (IRCCS), Rome, Italy (practice no. 3839/2026, approved on 29 April 2026; transmission protocol no. 163 of 19 May 2026).

### 2.1. Clinical History and Imaging

A 10-year-old boy (42 kg, 150 cm; BMI 18.67 kg/m^2^) with CMMRD was referred for surgical treatment of a recurrent left frontal astrocytoma. His medical history was notable for stage III right laterocervical Burkitt lymphoma previously treated with chemotherapy and gastrointestinal adenomatous lesions consistent with his underlying cancer-predisposition syndrome.

Approximately one year before the present procedure, he had undergone resection of a left frontal brain lesion. Histopathological examination confirmed an IDH-mutant astrocytoma, WHO grade 3, according to the 2021 WHO Classification of Tumors of the Central Nervous System [8]. After surgery, he received four cycles of compassionate-use immunotherapy.

During follow-up, serial brain magnetic resonance imaging (MRI) showed progression of the left frontal lesion, in close proximity to cortical regions involved in language function (Figure 1).

Neurological examination revealed no focal deficits, with preserved language comprehension and production. Electroencephalography showed organized cerebral activity without epileptiform abnormalities.

Given the lesion location and the need to preserve language function, advanced neuroimaging and functional assessments were performed to better characterize the relationship between the tumor and eloquent cortex. Imaging findings suggested involvement of the dominant frontal language network, raising concerns about potential postoperative deficits with conventional tumor resection. Based on these findings, the neurosurgical team considered awake craniotomy with intraoperative functional cortical mapping as the preferred surgical strategy to maximize safe tumor resection while preserving neurological function.

### 2.2. Multidisciplinary Preoperative Evaluation

The patient underwent a comprehensive multidisciplinary preoperative evaluation—neurosurgical, neuropsychological, and anesthesiologic. As part of our institutional pathway, candidate patients undergo dedicated neuropsychological assessment of developmental maturity, ability to cooperate during intraoperative testing, and performance on the language and cognitive tasks used for cortical mapping; once considered suitable, they are introduced to the operating-room environment before surgery to familiarize themselves with the setting and staff. On this assessment, the patient demonstrated adequate emotional maturity for his age, good engagement with the healthcare team, and satisfactory task performance, with a highly involved and supportive family.

A pediatric anesthesiologist met with the patient and his family on multiple occasions before surgery, explaining the anesthetic plan and the awake craniotomy procedure in age-appropriate terms. The patient showed good attention, understanding of the procedure, and appropriate responses during simulation of intraoperative tasks, supporting his suitability for awake craniotomy.

### 2.3. Preoperative Anesthesiologic Assessment

Preoperative anesthesiologic assessment classified the patient as American Society of Anesthesiologists (ASA) physical status III. He had a history of previous transfusions and a mild preoperative anemia (hemoglobin 9.2 g/dL), considered multifactorial and consistent with anemia of chronic disease in the context of the underlying malignancy and ongoing oncological treatment, with a contributing inflammatory component following a recent, resolved respiratory infection. The anemia was chronic, stable on serial controls, and clinically well tolerated, and no specific preoperative correction was undertaken; in view of the expected duration and potential blood loss of the procedure, type-specific packed red blood cells were made available in the operating room according to institutional practice. No known drug allergies were reported. The patient was receiving prophylactic levetiracetam, started for electroencephalographic abnormalities in the absence of clinical seizures and continued throughout the perioperative period. Preoperative airway assessment predicted no difficult airway: Mallampati class I, mouth opening ≥3 cm, thyromental distance ≥6 cm, and full, painless neck mobility, with normal dentition. Given the expected prolonged duration of surgery, the possibility of intraoperative blood loss, the need for repeated neurological assessment during the awake phase, and the complexity of airway management in the lateral position, an asleep–awake–asleep anesthetic strategy was planned in advance.

According to our institutional practice for pediatric awake craniotomy, anesthetic care was provided by two pediatric anesthesiologists from the beginning of the procedure: one with specific experience in neuroanesthesia, responsible for anesthetic drug administration, physiological monitoring, and ventilatory management during the asleep phases; and the other with expertise in airway management, positioned in direct line of sight of the patient and working in close coordination with the neuropsychologist, particularly during awakening, the awake phase, and airway transitions.

### 2.4. Induction of Anesthesia and Airway Management

On the day of surgery, the patient was appropriately fasted and arrived in the operating room with a functioning peripheral intravenous line. He appeared calm and was accompanied by his father until induction of anesthesia. Standard monitoring was applied, including electrocardiography, noninvasive blood pressure monitoring, and pulse oximetry. Antiemetic and gastric prophylaxis were administered with ondansetron 5 mg and omeprazole 30 mg.

General anesthesia was induced with propofol 150 mg (3.6 mg/kg), fentanyl 100 µg (2.4 µg/kg), and rocuronium 50 mg (1.2 mg/kg). The fentanyl dose, although at the higher end of the pediatric range, was chosen to attenuate the hemodynamic and intracranial-pressure response to fiberoptic-guided intubation and Mayfield three-pin fixation; given as a single bolus several hours before the awake phase, it was not expected to impair intraoperative cooperation. Rocuronium was administered as a single intubating dose, with no subsequent neuromuscular blockade. Quantitative neuromuscular monitoring (train-of-four at the adductor pollicis) was used throughout to guide management of neuromuscular function. Induction was performed gradually in a calm environment, with background music requested by the patient and in the presence of the parent, who left the operating room once the child was asleep.

According to our institutional protocol for pediatric awake craniotomy, an air-Q intubating laryngeal mask airway (size 3) was first inserted to secure the airway. A single-use flexible bronchoscope (Ambu^®^ aScope™ 4 Broncho Regular; Ambu A/S, Ballerup, Denmark) was then advanced through the device to visualize the glottis, and a 6.0 mm cuffed endotracheal tube was railroaded over the bronchoscope into the trachea once the vocal cords were clearly identified.

This fiberoptic-guided approach through a supraglottic conduit was deliberately preferred over direct laryngoscopy to achieve an atraumatic, visually confirmed intubation that minimizes the pressor response to airway instrumentation and the associated rise in intracranial pressure in a child with a recurrent space-occupying lesion, and—importantly—to standardize, under optimal initial conditions, the same airway technique that would later be required for reintubation in the lateral position after the awake phase, when direct laryngoscopy would have been markedly more challenging, thereby providing greater certainty and reliability for the lateral-position reintubation.

Correct tracheal placement was confirmed by continuous capnography and bilateral chest auscultation. Mechanical ventilation was subsequently initiated.

The air-Q was used exclusively as an intubating conduit and was removed immediately after correct tube placement had been confirmed.

During the initial asleep phase, after the manual induction bolus, anesthesia was maintained with propofol administered by target-controlled infusion (Paedfusor model). To avoid excessive dosing, the infusion was introduced gradually at the maintenance target (3–4 µg/mL) once the airway had been secured, and titrated under continuous bispectral index (BIS) and neurophysiological guidance; this allowed a smooth, controlled transition from the induction bolus to maintenance without risk of overdose. Remifentanil infusion (≤0.25 µg/kg/min) was adjusted according to hemodynamic responses.

### 2.5. Intraoperative Monitoring and Supportive Measures

After induction, additional invasive monitoring and vascular access were established. A right radial arterial catheter was placed to allow continuous beat-to-beat blood pressure monitoring and repeated arterial blood gas analysis during prolonged neurosurgery, particularly in view of the anticipated anesthetic transitions, potential blood loss, and the need for transfusion guidance. A 5.5 Fr central venous catheter was inserted into the left innominate vein under sterile conditions with intraoperative endocavitary electrocardiography confirmation, in order to secure reliable venous access for prolonged infusion therapy and perioperative hemodynamic management during a long and technically demanding procedure performed in the lateral position. A nasogastric tube was also inserted after induction to allow gastric decompression during prolonged surgery, reduce gastric distension after airway instrumentation and positive-pressure ventilation, and facilitate perioperative management in the event of postoperative delayed awakening or nausea.

Comprehensive intraoperative monitoring included electrocardiography, pulse oximetry, invasive arterial blood pressure, rectal temperature, central venous pressure, and BIS.

Intraoperative neurophysiological monitoring (IONM) was tailored to the procedure and comprised electroencephalography (EEG), electrocorticography (ECoG), and somatosensory evoked potentials (SSEPs), supervised in real time by a dedicated team—an experienced neurophysiology technologist and a clinical neurophysiologist—in the operating room. Multichannel EEG was recorded through subdermal needle electrodes arranged according to the International 10–20 System and adapted to the surgical field, allowing real-time detection of spontaneous epileptiform activity and stimulation-induced after-discharges. Four-limb SSEPs, obtained by bilateral stimulation of the median and posterior tibial nerves, were used to monitor the integrity of the ascending somatosensory pathways. Motor evoked potentials were intentionally omitted to streamline the protocol and facilitate the subsequent awake language-mapping phase.

Antibiotic prophylaxis with cefazolin 1.3 g was administered 30 min before skin incision and then every 4 h at the same dose throughout the procedure; osmotic therapy with mannitol 18 g was also given before incision. Intravenous fluids were maintained at 4 mL/kg/h throughout the procedure.

As the surgical plan for this lesion required the right lateral position, the patient was positioned accordingly, and the head was secured using Mayfield three-pin fixation (Figure 2).

### 2.6. Scalp Block and Local Anesthetic Infiltration

After induction of general anesthesia and before pin fixation and skin incision, a bilateral scalp nerve block was performed using a landmark-based technique targeting the supraorbital, supratrochlear, auriculotemporal, greater occipital, and lesser occipital nerves. Following careful aspiration before each injection, a mixture of mepivacaine and ropivacaine with epinephrine (1:200,000) was administered in fractionated aliquots on both sides, delivering mepivacaine 100 mg (2.4 mg/kg) and ropivacaine 37.5 mg (0.9 mg/kg). Additional field infiltration of the surgical site and pin sites was performed by the surgeon with lidocaine and ropivacaine with epinephrine (1:200,000), delivering lidocaine 80 mg (1.9 mg/kg) and ropivacaine 16 mg (0.4 mg/kg). The cumulative doses were therefore mepivacaine 100 mg (2.4 mg/kg), lidocaine 80 mg (1.9 mg/kg), and ropivacaine 53.5 mg (1.3 mg/kg). Each agent remained below the recommended pediatric maximum doses with epinephrine, and the cumulative exposure was within safe limits even accounting for the additive systemic toxicity of co-administered local anesthetics [9,10].

### 2.7. Awake Phase, Language Mapping, and Reinduction

Intraoperative image-guided neuronavigation, based on preoperative computed tomography (CT) and MRI data integrated into a three-dimensional reconstruction of the patient’s brain, was set up at the beginning of the procedure and used throughout surgery to assist anatomical orientation and guide tumor resection (Figure 3).

After completion of the craniotomy and approximately 45 min before the anticipated start of cortical mapping, the anesthetic regimen was progressively adjusted to facilitate a smooth transition to the awake phase. During this transition, propofol target-controlled infusion was gradually tapered and then discontinued, so that by the start of mapping the patient was fully awake, while remifentanil was continued at a low dose (0.03–0.05 µg/kg/min). At the same time, dexmedetomidine was started as a continuous infusion at 0.1 µg/kg/h and titrated up to 0.2 µg/kg/h to maintain light sedation while preserving spontaneous ventilation and cooperation. No loading dose was administered, to avoid the hemodynamic effects of bolus administration. The infusion was titrated dynamically, guided by the patient’s clinical response, interaction with the team, and the monitoring described above, to maintain a Richmond Agitation-Sedation Scale (RASS) score between −1 and 0 [11], compatible with reliable participation in language testing. In this way, dexmedetomidine served as a bridging sedative during progressive emergence from propofol anesthesia. Because rocuronium had been administered only as a single intubating dose at induction, several hours earlier, complete neuromuscular recovery had occurred spontaneously by the time of the awake phase; this was confirmed objectively by a train-of-four ratio ≥0.9 at the adductor pollicis, so that no pharmacological reversal was required. Before removal of the tube, while breathing spontaneously with minimal pressure support, the patient had normal respiratory parameters (respiratory rate approximately 18 breaths/min, tidal volume about 300 mL [~7 mL/kg], end-tidal CO_2_ 35–45 mmHg, kept within the normocapnic range to avoid cerebral vasodilation and raised intracranial pressure). The endotracheal tube was then removed, and the patient remained eupneic and hemodynamically stable, with SpO_2_ 98–100% on room air and no need for supplemental oxygen; an arterial blood gas analysis obtained after removal of the tube showed no respiratory gas derangement and a normal acid–base balance. This allowed transition to the awake phase, during which the patient’s face was left exposed and directly accessible, with surgical drapes tented and raised to allow unrestricted airway access for the dedicated anesthesiologist. A predefined airway rescue plan was also in place: should airway compromise have occurred, the team was prepared to reintubate immediately using the same air-Q-guided fiberoptic sequence standardized at induction—with the supraglottic device as the first-line rapid rescue tool—while the neurosurgical team stood ready to release the drapes and free access to the head without delay, and reconversion to general anesthesia remained available at any time.

During this emergence phase, dural opening and preparation of the surgical microscope were completed, while the anesthesiologist and the neuropsychologist gently supported the patient through awakening. The only event during the transition was a brief, self-limiting episode of agitation, age-consistent and unaccompanied by desaturation or hemodynamic instability, which settled promptly with reassurance alone and without pharmacological intervention.

Once fully responsive, the patient participated in intraoperative language mapping, performed using the classical Penfield technique with a 50 Hz bipolar stimulus train and concurrent EEG to detect stimulation-induced after-discharges. Stimulation reproducibly elicited transient speech arrest at two distinct sites within the dominant perisylvian language cortex, allowing the surgical team to mark these eloquent sites and delineate the functional boundary between the tumor and eloquent cortex. During mapping, the patient performed standardized object-naming and repetition tasks—counting from one to ten and naming images (including a snake, bell, airplane, and knife)—while conversational interaction about his dog was used to facilitate spontaneous speech and maintain engagement. To prevent oral dryness during prolonged speech testing, small amounts of melon-flavored ice pop were administered intermittently to lubricate the oral cavity and improve patient comfort (Figure 4).

In line with best clinical practices for awake craniotomy, the operating room team continuously used reassuring and encouraging language to reduce intraoperative distress. Positive reinforcement (e.g., “You are doing very well” or “Great job staying still and relaxed”) was provided throughout the awake phase, while negative or anxiety-provoking statements were intentionally avoided. After completion of language mapping, the patient was returned to general anesthesia and reintubated in the right lateral position to complete tumor resection in areas not adjacent to eloquent cortex or critical white matter tracts. Reintubation used the same sequence employed at induction—air-Q (size 3), fiberoptic visualization of the glottis, and railroading of a cuffed endotracheal tube over the bronchoscope. Having standardized this technique under optimal initial conditions, the team reproduced it reliably in the more demanding lateral position, and the procedure proceeded uneventfully in a fully awake, cooperative patient, without respiratory or hemodynamic instability.

Propofol target-controlled infusion had been used during the mapping phase for its rapid, predictable offset before the awake phase; once mapping was completed, these requirements no longer applied—also because the patient was not to be extubated in the operating room—and propofol was therefore discontinued in favor of sevoflurane, a simpler and stable option for the final phase. Remifentanil was continued for analgesic support.

Hemostasis and surgical closure were then completed without complications. Intraoperative blood gas analysis guided transfusion of two units of packed red blood cells. EEG and serial four-limb SSEP recordings were maintained until the end of the procedure, with no significant neurophysiological changes, confirming preservation of the monitored pathways. The overall duration of the procedure was approximately 15 h, reflecting the complexity of recurrent-tumor surgery and its three distinct phases (exposure, mapping-guided resection in the eloquent area, and final hemostasis and closure).

### 2.8. Predefined Intraoperative Safety and Conversion Plan

Before surgery, the multidisciplinary team agreed on a predefined safety plan for the awake phase. Rather than rigid numerical thresholds, shared clinical criteria were established to guide intervention and, if necessary, conversion to an asleep technique. Although none of these contingencies occurred, the plan was as follows:

- **Loss of cooperation or escalating anxiety:** First-line management by the dedicated anesthesiologist and the neuropsychologist through reassurance and re-engagement, drawing on the preoperative familiarization; conversion to an asleep technique reserved for sustained, irreversible loss of cooperation precluding reliable testing.
- **Stimulation-induced seizures:** Immediate cessation of cortical stimulation and irrigation of the cortex with ice-cold saline, with low-dose propofol or a benzodiazepine reserved for seizures persisting despite these measures.
- **Hypoventilation, airway obstruction, or desaturation:** Simple airway maneuvers and titration of sedation, escalating, if needed, to the predefined airway-rescue and reintubation pathway.
- **Aspiration risk:** Mitigated by gastric decompression through the nasogastric tube and by the immediately available reintubation pathway for definitive airway protection.
- **Failed or unreliable mapping:** Completion of the resection under general anesthesia without functional mapping.

Across all these scenarios, conversion to general anesthesia was regarded not as a complication but as a predefined, safe fallback. This is consistent with reported failure criteria for awake craniotomy, in which loss of intraoperative communication and uncontrolled seizures are the leading causes of conversion.

### 2.9. Postoperative Analgesia

Postoperative analgesia was provided through a multimodal regimen tailored to age and to the intracranial nature of the procedure. Intravenous acetaminophen 15 mg/kg every 6 h was administered around the clock as the baseline nonopioid component. To control background pain during the early postoperative phase, including the initial stay in the pediatric intensive care unit (PICU), a continuous intravenous infusion of morphine was started at 10 µg/kg/h (0.01 mg/kg/h) and titrated according to clinical response and Numeric Rating Scale (NRS) scores, with additional intravenous morphine boluses of 50 µg/kg available as rescue therapy for breakthrough pain. Systemic nonsteroidal anti-inflammatory drugs were withheld during the first 24 h after craniotomy because of the potential bleeding risk in the surgical bed; oral ibuprofen 10 mg/kg every 8 h was subsequently introduced, after agreement with the neurosurgical team, to reinforce nonopioid analgesia and facilitate opioid weaning. Pain scores remained below 4 on the NRS throughout the documented postoperative observation period, and no opioid-related adverse events were recorded.

### 2.10. Postoperative Course

At the end of surgery, the patient was transferred intubated to the PICU for close postoperative monitoring. Delayed extubation was a deliberate, planned decision rather than the consequence of any adverse event: after an approximately 15 h procedure for recurrent-tumor surgery, in line with our institutional practice for major prolonged surgery, extubation was performed in the controlled PICU environment once full respiratory and hemodynamic stability had been confirmed. The patient was extubated a few hours after admission, without complications and without apparent neurological deficits, and was transferred to the neurosurgical ward on postoperative day 1. Postoperative neurological evaluation demonstrated preserved language function, no focal neurological deficits, and progressive recovery of ambulation, with a Karnofsky Performance Status >70% [12]. The patient was discharged home on postoperative day 6. At the 1-month follow-up visit, he remained symptom-free, with no psychiatric or neuropsychological issues reported.

A short summary of the anesthetic management planning is provided in Figure 5.

## 3. Discussion

This case shows that pediatric awake craniotomy can be successfully performed in a carefully selected child when anesthetic management is embedded within a structured multidisciplinary pathway. In our 10-year-old patient with CMMRD, recurrent left frontal astrocytoma adjacent to eloquent language cortex, and preserved baseline language function, the asleep–awake–asleep strategy allowed effective intraoperative language mapping, completion of tumor resection, and an uncomplicated postoperative course without new neurological deficits. Beyond the rarity of the procedure in childhood, the clinical relevance of this case lies in the combination of recurrent tumor surgery, language mapping, and coordinated perioperative management in a pediatric setting.

The profile of our patient is consistent with the available pediatric literature. Bhanja et al., in a systematic review, reported that awake craniotomy remains uncommon but increasingly feasible in selected children, particularly between 7 and 17 years of age undergoing tumor resection near eloquent cortex [2]. Our case reflects this pattern and supports the concept that age alone should not be considered an exclusion criterion. Rather, suitability appears to depend on developmental maturity, preserved communication skills, emotional regulation, and family support.

This interpretation is further supported by Alcaraz García-Tejedor et al., who demonstrated high feasibility and acceptable morbidity when pediatric awake craniotomy is implemented within a structured multidisciplinary framework [5]. In our case, the favorable intraoperative course likely depended less on any single anesthetic maneuver than on the combination of preoperative neuropsychological assessment, repeated family-centered discussions, operating-room familiarization, and close collaboration among anesthesiologists, neurosurgeons, and neuropsychologists. This reinforces the idea that pediatric awake craniotomy should be regarded as a perioperative process rather than solely an intraoperative technique.

From an anesthetic perspective, the asleep–awake–asleep technique remains the most commonly reported approach in pediatric awake craniotomy and is typically based on propofol and short-acting opioids during the asleep phases, with increasing use of dexmedetomidine to facilitate a smoother awake phase [2,6]. Alternative strategies based on conscious sedation or monitored anesthesia care have also been described and may be appropriate in selected patients [3,13]. However, the pediatric literature remains limited [2,3,13], and the choice of anesthetic strategy should primarily reflect patient characteristics, expected duration of surgery, the functional goals of mapping, and the experience of the treating team.

Beyond the selection of anesthetic agents, advances in target-controlled infusion technology may further refine the management of these procedures. Although propofol administration in our case was based on the pediatric Paedfusor model [14], recently developed general-purpose pharmacokinetic–pharmacodynamic (PK/PD) models for propofol, remifentanil, and dexmedetomidine have been designed for use across a broad range of ages and body weights [15,16]. By providing a unified framework for drug titration from childhood to adulthood, these models may facilitate the delicate transitions between general anesthesia and responsive sedation that characterize both monitored anesthesia care and the asleep–awake–asleep technique, although their role in pediatric awake craniotomy remains to be established [15,16].

The available literature also suggests that pediatric awake craniotomy can be successfully performed using different practical intraoperative approaches. In the educational review by Henry et al., which included an illustrative pediatric case, the early phases were managed under spontaneous ventilation, with subsequent airway instrumentation later in the procedure [3]. By contrast, Cabral et al., in a recent case report of a 12-year-old child with recurrent ganglioglioma, described an asleep–awake–asleep strategy supported by dexmedetomidine, with successful completion of mapping and an uneventful postoperative course [17]. In our case, the same asleep–awake–asleep logic was combined with a more controlled airway approach consistent with institutional practice, allowing airway management under controlled conditions and facilitating planning for the later reintubation phase in the lateral position.

Although conscious sedation throughout the procedure may be attractive because of its simplicity and preservation of spontaneous breathing, it may also leave a narrower margin for intervention if respiratory depression, apnea, agitation, or an unexpected neurosurgical event occurs during the awake phase [2,3,5]. In this setting, airway rescue in the lateral position may be technically demanding, particularly in a child. Supporting this concern, Gruenbaum et al., in a cadaveric simulation model of awake craniotomy in the lateral position with three-pin fixation, found that supraglottic airway placement was the fastest and most reliable primary rescue technique, with fiberoptic intubation through the device as a practical backup [18]. Similarly, Tang et al. emphasized that emergency airway management is among the most demanding scenarios in awake craniotomy, because head fixation, draping, and lateral positioning may severely restrict airway access, while spontaneous ventilation must be preserved with little room for ventilatory intervention [19]. Taken together, these observations suggest that, in selected pediatric patients, airway management should be anticipated as part of the overall perioperative plan rather than viewed only as a rescue issue—consistent with Natalini et al., who, given the heterogeneity of the evidence, argued against claiming clear superiority for either asleep–awake–asleep or monitored anesthesia care [20]. On this basis, in the present case we secured a definitive cuffed endotracheal airway rather than maintaining a supraglottic airway as the primary airway throughout, reflecting the specific demands of the procedure: an approximately 15 h operation with two prolonged phases of controlled ventilation in the lateral position under three-pin fixation, in which a supraglottic primary airway would have carried a substantial risk of displacement, loss of seal, hypoventilation, and aspiration, with difficult rescue once the head was fixed and draped. The supraglottic device was therefore deliberately repurposed from “primary airway” to “intubation conduit and rescue tool,” providing a stable definitive airway during the asleep phases while preserving a rapid rescue pathway for the high-risk lateral reintubation. In our experience, this protocolized strategy offered the most favorable balance between airway safety and the functional demands of awake mapping, and we regard it as a reasonable default for prolonged pediatric awake craniotomy in the lateral position when the necessary expertise is available.

In our patient, this individualized approach extended beyond anesthetic drug selection to the broader perioperative organization: low-dose dexmedetomidine and remifentanil were integrated with prior scalp block and local infiltration, structured behavioral support, and continuous interaction among the anesthesiologist, neuropsychologist, and surgical team. This multimodal management was particularly important in maintaining cooperation and comfort during language testing while minimizing distress.

In line with prior observations, no seizures, major airway complications, or other major intraoperative adverse events occurred in our case [2,3]. In keeping with this, Akavipat et al., in a systematic review and meta-analysis, found a lower rate of intraoperative seizures in dexmedetomidine-based awake craniotomy regimens than in non-dexmedetomidine regimens [6].

Dexmedetomidine has become the sedative agent of choice for awake craniotomy, and pediatric reviews and series consistently report it as the cornerstone of asleep–awake–asleep and conscious-sedation approaches in children [2,3,4,5,6]. Its suitability rests on a favorable pharmacological profile: as a selective α2-adrenergic agonist acting through the locus coeruleus, it produces an arousable, NREM-sleep-like sedation with minimal respiratory depression and limited interference with cortical function and neurophysiological monitoring, so that, at low infusion rates, language and cognitive performance are preserved [21]. Echoing reports specifically addressing awake craniotomy in eloquent areas, we deliberately omitted the loading dose to limit cardiovascular depression and the hemodynamic oscillations associated with bolus administration—particularly relevant in the neurosurgical patient, in whom intraoperative hypertension may promote bleeding and edema and hypotension may impair cerebral perfusion—favoring instead a low-dose, dynamically titrated infusion [21,22,23]. Although dexmedetomidine is off-label in children—in the European Union it is approved only for sedation of adults in intensive care—this reflects the situation across Europe rather than a local exception, and its pediatric use is widespread and commonly governed by local or national protocols [24,25]. Although pediatric awake neurosurgery is less represented in the literature than the adult setting, the accumulated clinical experience supports the efficacy and safety of dexmedetomidine in children [2,3,4,5,6]. In the present case, it was used within our established institutional practice, and written informed consent was obtained from the parents, explicitly covering the possible use of off-label medications and acknowledging that such use is supported by the available literature as effective and safe.

Another important aspect highlighted by this case is the central role of team coordination. Psychological preparation and familiarization with the operative environment are recognized as key elements of pediatric awake brain surgery [3,7]. In our case, these factors were complemented by continuous intraoperative communication and by the presence of a dedicated multidisciplinary team able to support both the technical and relational demands of the awake phase. This may be especially relevant in children, in whom loss of cooperation, anxiety, or sensory overload may compromise mapping reliability even in the absence of overt anesthetic or surgical complications [3,7,19].

This case report is inherently limited by its single-patient design, which restricts generalizability and carries an unavoidable risk of selection and interpretation bias. Beyond the single-patient design, the favorable outcome should also be read in context: it reflected not only careful patient selection but also institutional prerequisites that may not be uniformly available—an established protocol for pediatric awake craniotomy, an experienced multidisciplinary team (pediatric neuroanesthesiologists, neurosurgeons, neurophysiology, and neuropsychology), and the capacity to manage the airway as an emergency in a restricted surgical field. Reproducibility therefore depends on local expertise and resources, and centers considering a similar approach should ensure both appropriate patient selection and these capabilities. Its main strengths are the detailed description of perioperative planning in a rare pediatric awake craniotomy, the emphasis on multidisciplinary coordination, and the documentation of preserved postoperative neurological and neuropsychological status at short-term follow-up.

## 4. Conclusions

This case illustrates that, in carefully selected pediatric patients managed within a structured multidisciplinary pathway, awake craniotomy can be performed safely, and it adds to the still limited body of pediatric evidence. In our patient, an individualized asleep–awake–asleep strategy allowed successful intraoperative language mapping, tumor resection, and preservation of postoperative neurological and neuropsychological function. Beyond the technical aspects of anesthesia, this experience underscores that the favorable outcome rested not on any single intraoperative technique but on rigorous patient selection, structured preoperative preparation, and close multidisciplinary coordination throughout the perioperative course. Given its single-patient nature, these observations are hypothesis-generating and should be interpreted with caution; further pediatric evidence is needed to refine selection criteria, confirm these findings in larger prospective series, and define best perioperative practices.

## Figures and Tables

**Figure 1:**
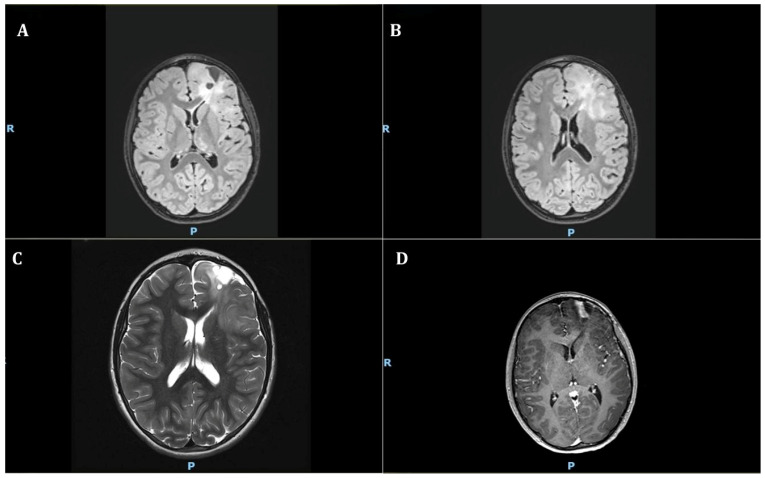
Preoperative brain magnetic resonance imaging showing the recurrent left frontal/fronto-insular lesion adjacent to eloquent cortical areas. (**A**,**B**) Axial FLAIR images demonstrating extension of the lesion toward the high insular and left fronto-insular regions. (**C**) Axial T2-weighted image showing signal abnormality corresponding to the recurrent lesion in the left fronto-insular region. (**D**) Axial contrast-enhanced T1-weighted image demonstrating the recurrent left frontal lesion in close proximity to cortical regions involved in language function. R, right; P, posterior.

**Figure 2:**
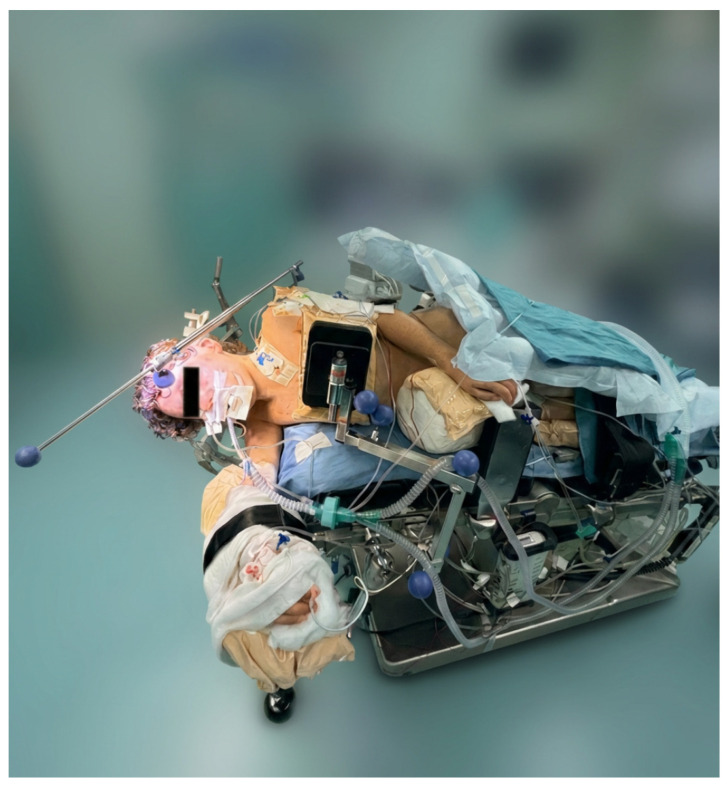
Patient in the right lateral position with head secured in a Mayfield three-pin head clamp.

**Figure 3:**
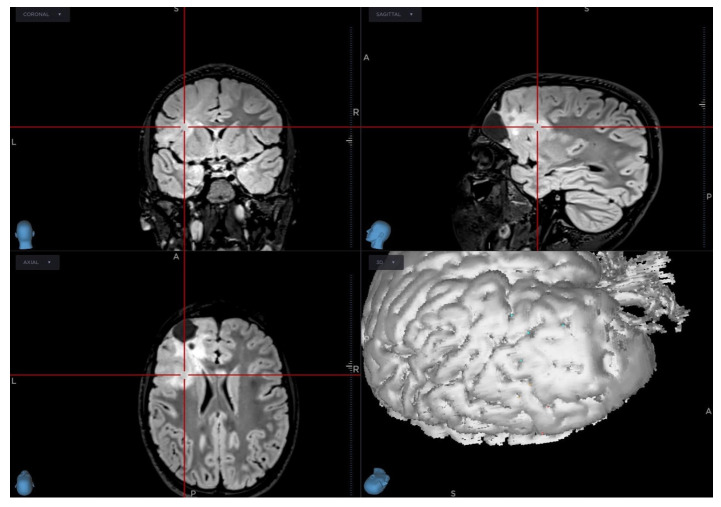
Preoperative neuronavigation planning based on integrated CT and MRI data, showing multiplanar reconstruction and three-dimensional visualization of the lesion. The system was used intraoperatively to assist anatomical orientation and guide tumor resection. The red lines/contours delineate the boundaries of the recurrent lesion (tumor localization).

**Figure 4:**
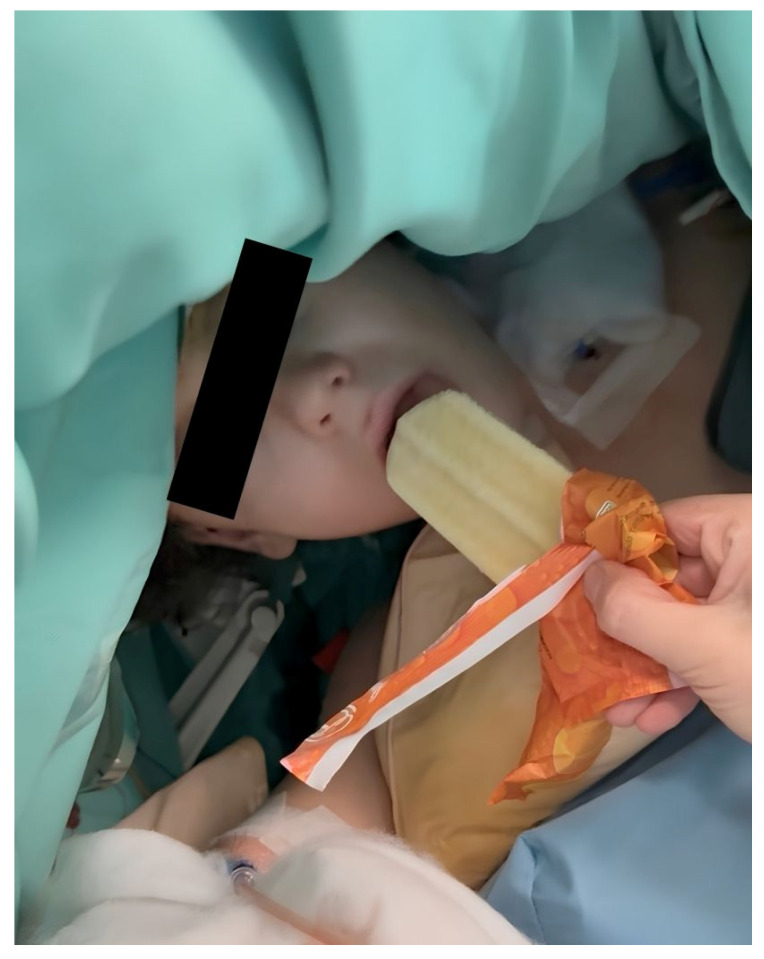
Melon-flavored ice pop administered during the awake phase to reduce oral dryness and improve comfort.

**Figure 5:**
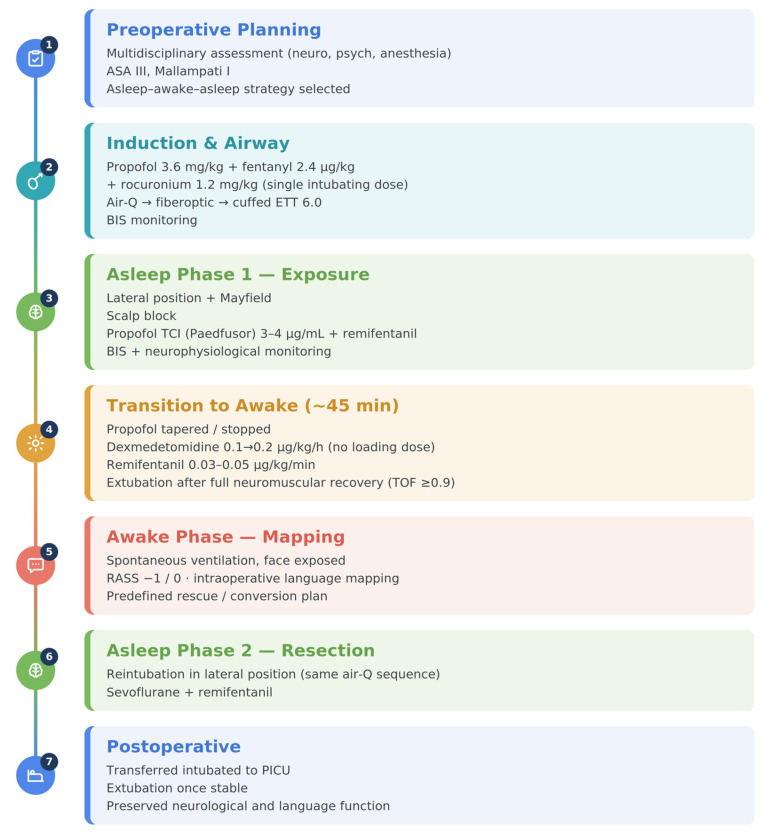
Short summary of the anesthetic management planning. Overview of the asleep–awake–asleep protocol across seven perioperative phases—from preoperative planning to postoperative course—summarizing the agents, airway approach, and monitoring in each phase. BIS, bispectral index; ETT, endotracheal tube; PICU, pediatric intensive care unit; RASS, Richmond Agitation-Sedation Scale; TCI, target-controlled infusion; TOF, train-of-four.

## Data Availability

The data presented in this study are available on request from the corresponding author due to privacy and ethical restrictions.
